# A Celebration of the Extraordinary Life of Late Professor Tatiana V. Serebrovskaya (Kyiv, Ukraine) in Advancing Hypoxia Science and Medicine

**DOI:** 10.1089/ham.2022.0046

**Published:** 2022-09-14

**Authors:** Erik R. Swenson, Robert T. Mallet, Lei Xi, Eugenia Manukhina, Fred Downey, Johannes Burtscher, Hannelore Ehrenreich, Martin Burtscher

**Affiliations:** ^1^ VA Puget Health Care System, University of Washington, Seattle, Washington, USA.; ^2^ Department of Physiology and Anatomy, University of North Texas Health Science Center, Fort Worth, Texas, USA.; ^3^ Pauley Heart Center, Division of Cardiology, Department of Internal Medicine, Virginia Commonwealth University, Richmond, Virginia, USA.; ^4^ Department of Biomedical Sciences, University of Lausanne, Lausanne, Switzerland.; ^5^ Clinical Neuroscience, Max Planck Institute for Multidisciplinary Sciences, Göttingen, Germany.; ^6^ Department of Sport Science, University of Innsbruck, Innsbruck, Austria.

**FIG. 1.**
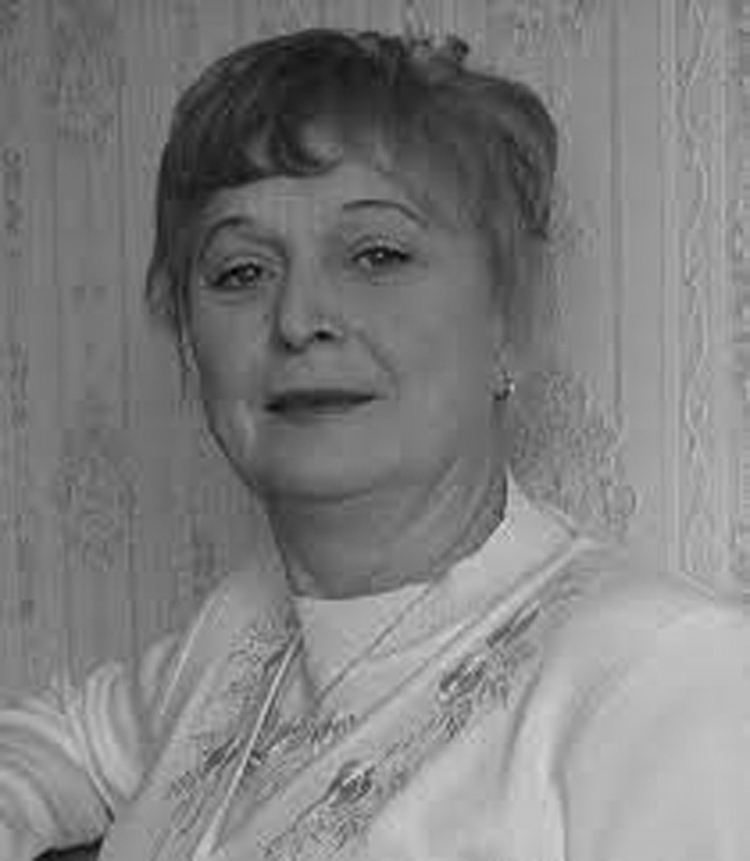
Professor Tatiana V. Serebrovskaya. Adopted from: https://altitudeclinic.com/blog/2021/07/dr-tatiana-serebrovskaya-memorial

One of Ukraine's leading scientists and innovators, our dear friend Professor Tatiana V. Serebrovskaya, passed away on April 5, 2021, from consequences of COVID-19. (Fig. 1).

Ironically, the aim of Tatiana's last article, which she coauthored with her daughter Zoya, was to contribute to a better mechanistic understanding of hypoxia and the cytokine storm in COVID-19 (Serebrovska et al, 2020). Tatiana was a highly respected researcher and principal scientist at the Department of Hypoxic States Investigation, Bogomoletz Institute of Physiology, National Academy of Sciences of Ukraine, Kyiv, Ukraine, a country presently tormented by an atrocious war and invasion by the Russian Federation.

**FIG. 2.**
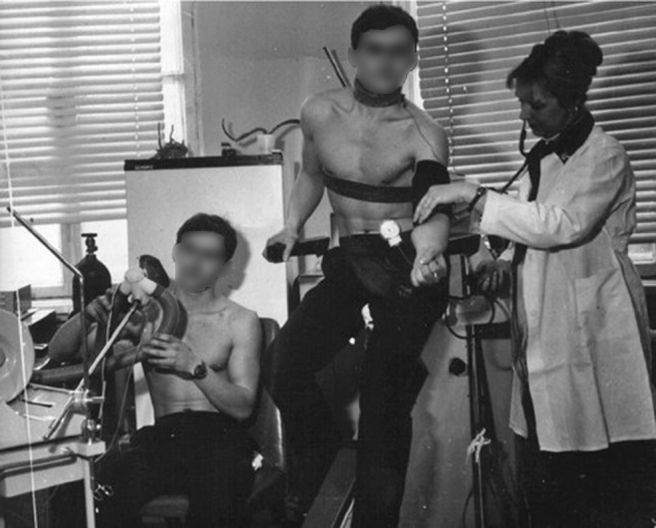
Tatiana in her young years, performing research in the exercise physiology lab. Adopted from Fig. 23.1 - “Twins investigation at the Bogomoletz Institute of Physiology, Kiev, Ukraine, 1979 (Researcher: Tatiana Serebrovskaya).” (Serebrovskaya and Xi, 2016).

**FIG. 3.**
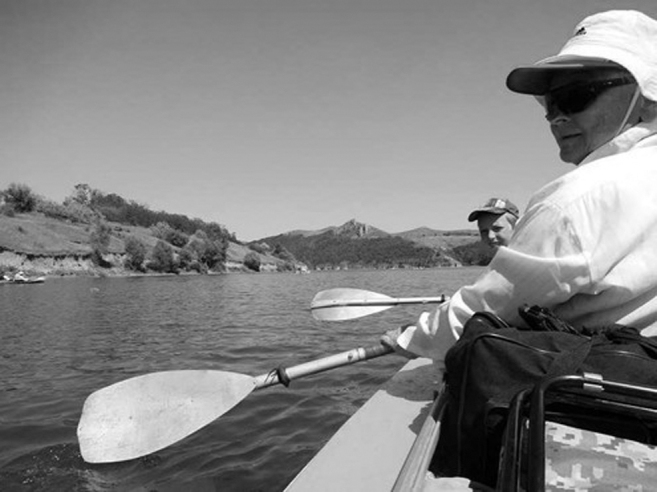
Tatiana, as a “captain” in her private life. Private photo: Gift from Tatiana Serebrovskaya to Hannelore Ehrenreich.

Tatiana was a highly admirable and exceptional human being, and even though it seems all her life was about science (Fig. 2), she was also a devoted captain in her private life (Fig. 3).

We want to specifically highlight Tatiana's life-long scientific passion to explore the health benefits of targeted or controlled ambient hypoxic exposures. Tatiana pioneered research on intermittent hypoxia applications to treat and prevent human diseases, a subject now receiving increasing attention and emerging as a fertile research field for the development of promising, safe, and noninvasive strategies to tackle numerous pathologies. Her seminal article “Intermittent hypoxia research in the former Soviet Union and the Commonwealth of Independent States: history and review of the concept and selected applications” (Serebrovskaya, 2002) introduced to this journal's global readership the groundbreaking accomplishments of scientists working on health-promoting hypoxia interventions in the former Soviet Union, but shrouded by the Iron Curtain.

Tatiana's tireless efforts allowed the successful translation of intermittent hypoxia results from the laboratory bench to the clinical bedside. Her productive enthusiasm resulted in >250 scientific articles (many in Ukrainian and Russian). Tatiana was awarded the medal for Honesty in Science by the International Association of Scientists, and was founding member and copresident of the Polish-Ukrainian Respiratory Working Group.

Among many other trailblazing discoveries, Tatiana and her colleagues demonstrated the beneficial effects of targeted ambient hypoxic exposures on the cardiorespiratory system and exercise performance in older people (Shatilo et al, 2008), the potential to prevent or treat systemic hypertension (Serebrovskaya et al, 2008), possible uses for immunotherapy (Serebrovskaya et al, 2011), provided a broad overview on hypoxia conditioning generally in human diseases (Xi and Serebrovskaya, 2012), putative important roles in the prevention and/or therapy of cardiovascular diseases (Serebrovskaya and Xi, 2016), and beneficial effects of hypoxia–hyperoxia on the development and/or progress of dementia (Mallet et al, 2020; Serebrovska et al, 2019).

Tatiana's pioneering research made her immortal. She will be a beacon for generations of future researchers working on the application of hypoxia-related interventions and her legacy will certainly bear abundant fruit for human health. We sincerely hope that the global scientific community's commitment enables Ukrainian scientists to continue their work in safety until the barbaric war comes to a hopefully very rapid end and allows Ukrainian science to thrive.
